# Adipose tissue stearoyl-CoA desaturase 1 index is increased and linoleic acid is decreased in obesity-prone rats fed a high-fat diet

**DOI:** 10.1186/1476-511X-12-2

**Published:** 2013-01-08

**Authors:** Jonathan Cedernaes, Johan Alsiö, Åke Västermark, Ulf Risérus, Helgi B Schiöth

**Affiliations:** 1 Department of Neuroscience, Uppsala University, BMC, Uppsala, SE, 75124, Sweden; 2 Department of Psychology, University of Cambridge, Cambridge, UK; 3 Department of Public Health and Caring Sciences, Clinical Nutrition and Metabolism, Uppsala University, Uppsala, Sweden

**Keywords:** Desaturase, Diet-induced obesity, Fatty acid composition, High-fat diet, Linoleic acid, Obesity prone, Obesity resistant, Subcutaneous adipose tissue, SCD-1, Stearoyl-CoA desaturase

## Abstract

**Background:**

Fatty acid (FA) composition and desaturase indices are associated with obesity and related metabolic conditions. However, it is unclear to what extent desaturase activity in different lipid fractions contribute to obesity susceptibility. Our aim was to test whether desaturase activity and FA composition are linked to an obese phenotype in rats that are either obesity prone (OP) or resistant (OR) on a high-fat diet (HFD).

**Methods:**

Two groups of Sprague–Dawley rats were given ad libitum (AL-HFD) or calorically restricted (HFD-paired; pair fed to calories consumed by chow-fed rats) access to a HFD. The AL-HFD group was categorized into OP and OR sub-groups based on weight gain over 5 weeks. Five different lipid fractions were examined in OP and OR rats with regard to proportions of essential and very long-chain polyunsaturated FAs: linoleic acid (LA), alpha-linolenic acid, eicosapentaenoic acid, docosahexaenoic acid and the stearoyl-CoA desaturase 1 (SCD-1) product 16:1n-7. FA ratios were used to estimate activities of the delta-5-desaturase (20:4n-6/20:3n-6), delta-6-desaturase (18:3n-6/18:2n-6), stearoyl-CoA desaturase 1 (SCD-1; 16:1n-7/16:0, SCD-16 and 18:1n-9/18:0, SCD-18), de novo lipogenesis (16:0/18:2n-6) and FA elongation (18:0/16:0). Fasting insulin, glucose, adiponectin and leptin concentrations were measured in plasma.

**Results:**

After AL-HFD access, OP rats had a significantly higher SCD-16 index and 16:1n-7 proportion, but a significantly lower LA proportion, in subcutaneous adipose tissue (SAT) triacylglycerols, as well as significantly higher insulin and leptin concentrations, compared with OR rats. No differences were found between the two phenotypes in liver (phospholipids; triacylglycerols) or plasma (cholesterol esters; phospholipids) lipid fractions or for plasma glucose or adiponectin concentrations. For the desaturase indices of the HFD-paired rats, the only significant differences compared with the OP or OR rats were higher SCD-16 and SCD-18 indices in SAT triacylglycerols in OP compared with HFD-paired rats.

**Conclusion:**

The higher SCD-16 may reflect higher SCD-1 activity in SAT, which in combination with lower LA proportions may reflect higher insulin resistance and changes in SAT independent of other lipid fractions. Whether a lower SCD-16 index protects against diet-induced obesity is an interesting possibility that warrants further investigation.

## Background

Obesity is a serious health concern that is increasing at an unprecedented rate worldwide. Excess intake of high-energy, highly palatable food may in turn result in a decreased metabolic response to increased fat intake, promoting a vicious cycle of weight gain [1]. However, even when presented with a high-energy diet, not all individuals become obese [2]. Thus, some are seemingly resistant to an obesity-inducing environment. Understanding the underlying mechanisms of this phenomenon is one of the central questions in the study of food intake regulation [3]. Just as humans differ in their susceptibility to diet-induced weight gain, individual animals within certain rodent strains also show different responses to excess energy intake [4-6]. On an ad libitum high-fat diet (HFD), the outbred Sprague–Dawley strain of rats can be divided into obesity-prone (OP) and obesity-resistant (OR) phenotypes to model human diet-induced obesity (DIO) [7,8]. This two-phenotype model can be useful for elucidating mechanisms that drive DIO, and to identify physiological and biochemical differences between the two groups.

Compared with their OR counterparts, OP rats show a more adverse metabolic risk profile as well as lower energy expenditure on a HFD [9,10], and a proadipogenic expression profile [11-13]. In addition, OP rats also have lower relative fat oxidation and fat oxidation enzyme expression [14-17]. However, the mechanisms behind these observed differences in DIO are still unclear, and further studies are needed to assess other potential metabolic and biochemical factors of importance.

Although OP/OR Sprague–Dawley rats represent key models for the study of differential susceptibility to DIO, the contribution of desaturase activities and fatty acid (FA) composition in various lipid fractions to these phenotypes has not been evaluated. Some differences in FA composition have been found between OP and OR animals, but the data are limited to a few FAs and lipid fractions [13], and not all desaturase indices have been studied.

Accumulating data suggest that the FA profile in plasma and metabolic tissues associates strongly with obesity and insulin resistance [18,19]. An altered FA metabolism and desaturase activity have been shown to be involved in body fat storage and oxidation [20,21]. Certain FAs and their ratios can also be utilized as important risk markers for various diseases and desaturase indices have been closely linked to several obesity-related conditions [22-26]. Especially, SCD-16 and D6D have been found to correlate positively with obesity and development of the metabolic syndrome [19,24,27]. The desaturase indices are FA product-to-precursor ratios that have been used to indirectly calculate the activities of the enzymes responsible for desaturating FAs [28]. By inserting double bonds into the FA carbon chains, desaturases convert saturated into unsaturated FAs, which in turn are substrates for various lipids and participate in signal transduction and food intake regulation [29,30]. The three desaturases, Δ5- (D5D), Δ6- (D6D) and, especially, the Δ9-desaturase (also known as stearoyl-CoA-desaturase or SCD-1), have been extensively studied (for review see Bjermo & Risérus [31]). SCD-1 is increased by saturated FAs and depressed by polyunsaturated FAs. Since high SCD-1 activity is associated with decreased fat oxidation and increased FA synthesis, and since an SCD-1 knockout leads to resistance to DIO, SCD-1 has been proposed as a potential target in the treatment of obesity [31,32].

Given the role for FA composition and desaturases in pathophysiology and metabolism we aimed to investigate these factors in response to HFD-induced weight gain. Potential differences between the OP and OR phenotypes in desaturase indices and biochemically important FAs, were investigated in five lipid fractions involved in FA handling and metabolism: subcutaneous adipose tissue triacylglycerols (SAT-TG), liver phospholipids (liver PL), liver triacylglycerols (liver TG), plasma cholesterol esters (PL-CE) and plasma phospholipids (PL-PL). In order to infer the impact of diet versus metabolic differences, we chose to study three groups: an ad libitum HFD group of rats divided into OP and OR, and a calorically restricted HFD group as control.

## Results

We used two dietary groups divided into a total of three experimental groups. One group of rats had ad libitum access to a HFD (AL-HFD, n = 24); a HFD-paired group (n = 10) were fed the HFD but were calorically restricted to follow the weight trajectory of male Sprague–Dawley rats fed regular chow. The rats in the AL-HFD group were further divided into either obesity-prone (OP, n = 12) or obesity-resistant (OP, n = 12), based on a median split of the gain in body weight (dBw) on the HFD after the 5-week experiment. There was no significant difference in initial body weight between the three groups.

**Figure 1:**
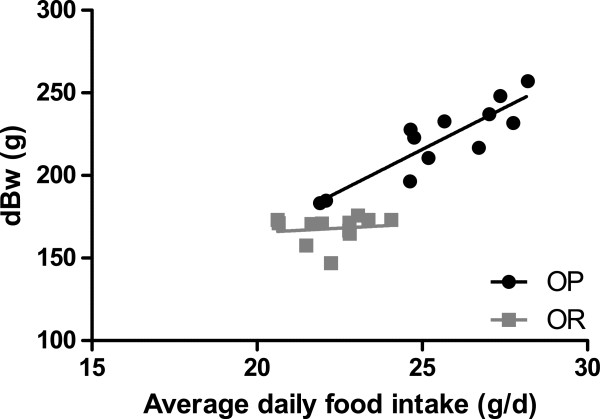
**Food intake was correlated with dBw only in OP rats. **Average daily food intake was highly correlated with gain in body weight (dBw) in obesity-prone (OP; r = 0.87, P < 0.001) but not in obesity-resistant rats (OR; r = 0.34, P = 0.28). Data analyzed using Spearman’s rank correlation.

After five weeks on a HFD, the obesity-prone (220.8 ± 23.5 g; P <0.001) and obesity-resistant (167.9 ± 8.2 g; P < 0.01) rats had gained significantly more weight than the calorically restricted HFD-paired (133.2 ± 21.7 g) group (Table 1). Cumulatively over the five-week period, OP rats ate more than OR rats (P < 0.001, data not shown). Average daily food intake was highly correlated with dBw in OP (r = 0.87, P < 0.001) but not in OR rats (r = 0.33, P = 0.28) (Figure 1). Food efficiency (dBw/average daily food intake in grams) was significantly higher (P < 0.001) in OP (8.65 ± 0.43) than in OR rats (7.56 ± 0.48).

**Table 1:** Body weight, food intake and fasting metabolic parameters for the three experimental groups of rats

	**OP**		**OR**		**HFD-paired**	
	**Mean**	**SD**	**Mean**	**SD**	**Mean**	**SD**
Initial BW (g)^a^	352.3	12.2	350.3	13.2	353. 2	16.8
Final BW (g)	573.1^###, †††^	29.9	518.2^‡‡^	12.3	486.4	17.3
Gain in BW (dBW) (g)^a^	220.8^###, ††^	23.5	167.9	8.2	133	21.7
Food consumption (g/day)	25.5^###, †††^	2.1	22.3^‡‡^	1.1	20.1	0.18
Energy consumption (kJ/day)	504.5^###, †††^	40.6	441.1^‡‡^	21	396.8	3.6
Insulin (μU/mL)^a^	127.80^#^^,^^††^	34.26	79.65	31.74	87.59	52.50
Glucose (mmol/l)	6.73	0.54	6.29	0.50	6.51	0.42
Leptin (ng/mL)	22.13^###, ††^	5.98	15.90	4.21	11.19	3.53
Adiponectin (μg/mL)	2.32^#^	0.51	1.94	0.34	1.82	0.44

Body weight (*BW*) and food intake in obesity-prone (*OP*), obesity-resistant (*OR*) and HFD-paired rats. The symbol ^#^ indicates a significant difference between OP and HFD-paired; ^†^ between OP and OR; ^‡^ between OR and HFD-paired. Level of significance: ^#^/^†^/^‡^, P < 0.05; ^##^/^††^/^‡‡^, P < 0.01; ^###^/^†††^/^‡‡‡^, P < 0.001. All tests were done using One-Way ANOVAs, followed by Tukey’s t-test when significant; or Kruskal Wallis test (for non-normally distributed data; indicated by a superscript “a” where performed), followed by Dunn’s multiple comparison test when significant.

### Desaturase indices in OP, OR and HFD-paired rats

**Figure 2:**
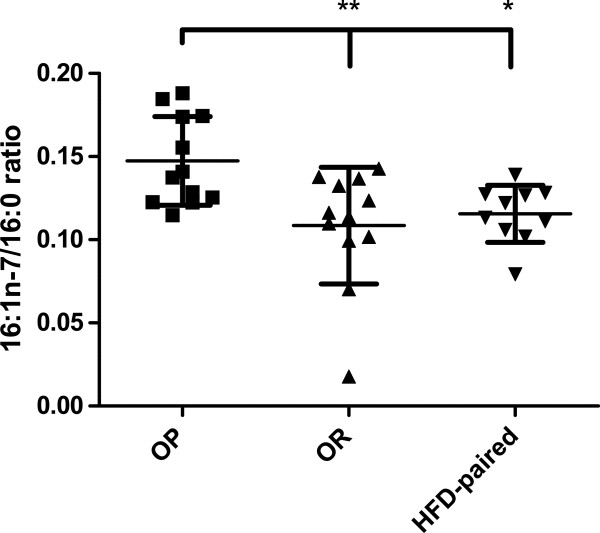
**SCD-16 in SAT-TG increased in OP rats fed a HFD. **HFD-induced SCD-16 activity calculated from the subcutaneous adipose tissue triacylglycerol (SAT-TG) ratio 16:1n-7/16:0. Obesity-prone (OP) rats had a significantly higher ratio than obesity-resistant (OR; P < 0.01) and HFD-paired (P < 0.05) rats, even after removing the lowest value (outlier) in OR. Scatter plot lines with error bars indicate means ± SD; data analyzed with One-way ANOVA and Tukey’s pos-hoc test.

Comparing the desaturase indices of OP and OR, we found a significant difference for SCD-16 in SAT-TG, with OP rats having a higher index than OR rats (P < 0.01) **(**Table 2; Figure 2**)**. Comparing OP and OR with HFD-paired rats, significant differences were detected in SAT-TG, where SCD-16 was significantly higher in OP compared with HFD-paired rats (P < 0.05), but no significant difference was seen between OR and HFD-paired rats. SAT-TG SCD-18 was significantly higher in OP compared to HFD-paired rats (P < 0.01), but no difference was detected between OR and HFD-paired rats. For all other tested desaturase indices in SAT-TG and the four other lipid fractions (PL-CE, PL-PL, liver PL and liver TG), no significant differences were found between OP, OR and HFD-paired rats.

**Table 2:** Fatty acid composition and FA enzyme indices in the three different groups of rats

**SAT-TG**						
	**OP**		**OR**		**HFD-paired**	
	**Mean**	**SD**	**Mean**	**SD**	**Mean**	**SD**
**SCD-16**	0.15^#, ††^	0.03	0.11	0.04	0.12	0.02
**SCD-18**	7.6^##^	0.7	7.0	0.7	6.5	0.6
**D6D**^a^	2.1 *10^-3^	0.6 *10^-3^	2.3 *10^-3^	0.9 *10^-3^	2.4 *10^-3^	1.3 *10^-3^
**D5D**	6.5	1.2	6.3	1.3	5.7	1.3
**DNL**	0.94	0.06	0.89	0.06	0.88	0.05
**EI**	0.22^##^	0.02	0.24	0.04	0.27	0.03
**16:1n-7**	3.43^#, ††^	0.67	2.49	0.83	2.63	0.44
**18:2n-6**	24.76^#, ††^	1.07	25.94	0.67	25.69	0.83
**18:3n-3**^a^	1.47^##^	0.07	1.47^‡‡^	0.12	1.35	0.05
**20:5n-3**	0.03	0.01	0.03	0.01	0.02	0.00
**22:6n-3**	0.10^#^	0.03	0.09	0.02	0.07	0.02
**PL-CE**						
	**OP**		**OR**		**HFD-paired**	
	**Mean**	**SD**	**Mean**	**SD**	**Mean**	**SD**
**SCD-16**	0.11	0.02	0.09	0.02	0.10	0.03
**SCD-18**	7.7	1.2	7.9	1.5	7.8	1.8
**D6D**	15 *10^-3^	2 *10^-3^	14 *10^-3^	3 *10^-3^	16 *10^-3^	4 *10^-3^
**D5D**	271	23	277	27	248	55
**DNL**^a^	0.43	0.06	0.40	0.03	0.40	0.05
**EI**	0.13	0.02	0.13	0.03	0.16	0.03
**16:1n-7**	0.75	0.12	0.65	0.12	0.74	0.22
**18:2n-6**^a^	16.77	1.43	17.80	1.67	18.04	2.76
**18:3n-3**	0.13^#^	0.03	0.13^‡^	0.03	0.17	0.04
**20:5n-3**	0.16	0.02	0.13^‡^	0.03	0.18	0.04
**22:6n-3**	1.37	0.14	1.47	0.21	1.27	0.20
**PL-PL**						
	**OP**		**OR**		**HFD-paired**	
	**Mean**	**SD**	**Mean**	**SD**	**Mean**	**SD**
**SCD-16**	0.012	0.002	0.011	0.003	0.011	0.003
**SCD-18**	0.19	0.03	0.21	0.03	0.22	0.03
**D6D**^b^	3.4 *10^-3^	0.8 *10^-3^	3.2 *10^-3^	0.4 *10^-3^	N/A	N/A
**D5D**	43	6	45	5	39	9
**DNL**	0.92	0.13	0.90	0.07	0.95	0.24
**EI**^a^	1.85^###^	0.19	1.74^‡‡^	0.24	1.53	0.061
**16:1n-7**	0.18	0.03	0.18	0.04	0.20	0.05
**18:2n-6**	17.76	2.95	18.50	1.80	20.06	3.33
**18:3n-3**^a^	0.08	0.02	0.06	0.02	0.09	0.03
**20:5n-3**^a^	0.08	0.01	0.09	0.01	0.10	0.02
**22:6n-3**	4.22^#^	0.46	4.18^‡^	0.74	3.48	0.62
**Liver PL**						
	**OP**		**OR**		**HFD-paired**	
	**Mean**	**SD**	**Mean**	**SD**	**Mean**	**SD**
**SCD-16**	0.013	0.004	0.011	0.003	0.011	0.004
**SCD-18**^a^	0.16	0.02	0.16	0.03	0.16	0.03
**D6D**^a^	12 *10^-3^	1 *10^-3^	11 *10^-3^	2 *10^-3^	12 *10^-3^	2 *10^-3^
**D5D**^a^	58	7	61	6	58	17
**DNL**	2.0	0.1	2.0	0.2	2.0	0.4
**EI**	1.9	0.1	1.9	0.2	1.7	0.1
**16:1n-7**	0.18	0.03	0.18	0.04	0.20	0.05
**18:2n-6**	7.90	0.78	7.85	1.02	8.93	1.61
**20:5n-3**	0.07	0.01	0.07	0.01	0.08	0.02
**22:6n-3**	7.84	0.96	8.51^‡‡^	1.23	7.08	0.79
**Liver TG**						
	**OP**		**OR**		**HFD-paired**	
	**Mean**	**SD**	**Mean**	**SD**	**Mean**	**SD**
**SCD-16**^a^	0.047	0.020	0.040	0.008	0.039	0.013
**SCD-18**	14	1	13	1	13	1
**D6D**	15 *10^-3^	2 *10^-3^	15 *10^-3^	2 *10^-3^	15 *10^-3^	2 *10^-3^
**D5D**	6.8	0.5	6.9	0.7	6.8	1.0
**DNL**^a^	1.1	0.3	1.0	0.1	1.0	0.1
**EI**^a^	0.10	0.00	0.11	0.01	0.10	0.01
**16:1n-7**^a^	1.28	0.66	1.01	0.22	1.03	0.36
**18:2n-6**^a^	25.12	2.99	25.90	1.67	26.39	2.05
**18:3n-3**	0.85	0.15	0.83	0.11	0.85	0.12
**20:5n-3**	0.18	0.06	0.17	0.04	0.17	0.03
**22:6n-3**^a^	1.15	0.43	1.16	0.42	1.02	0.35

Fatty acid (FA) composition and FA enzyme indices in five lipid fractions in obesity-prone (OP) and obesity-resistant (OR) rats with ad libitum HFD access (AL-HFD), and energy-restricted HFD-paired rats (HFD-paired). FAs are presented as relative percentages of total FAs within each lipid fraction. The symbol ^#^ indicates a significant difference between OP and HFD-paired; ^†^ between OP and OR; ^‡^ between OR and HFD-paired. Level of significance: ^#^/^†^/^‡^, P < 0.05; ^##^/^††^/^‡‡^, P < 0.01; ^###^/^†††^/^‡‡‡^, P < 0.001. N/A, not available. All tests were done using One-Way ANOVAs, followed by Tukey’s t-test when significant; or Kruskal Wallis test (for non-normally distributed data; indicated by a superscript “;a” where performed), followed by Dunn’s multiple comparison test when significant. Where data for only two groups was available, Student’s *t*-test was used (indicated by a superscript “;b” where performed) for normally distributed data.

### De novo lipogenesis and elongation indices in OP, OR and HFD-paired rats

In the initial ANOVA there was a significant difference in the de novo lipogenesis (DNL) index of SAT-TG, which did not however remain significant in the post-hoc test. In PL-CE, PL-PL, liver PL and liver TG there were no significant differences for the DNL indices.

The elongation index (EI) of SAT-TG was significantly lower in OP (P < 0.01) compared with HFD-paired rats. In PL-PL, the EI was significantly higher in both OP (P < 0.001) and OR (P < 0.01) compared with HFD-paired rats. In PL-CE, liver PL and liver TG, no significant differences between the three groups were found for the EI. Results for all the studied indices can be found in Table 2.

### Fatty acid proportions in OP, OR and HFD-paired rats

**Figure 3:**
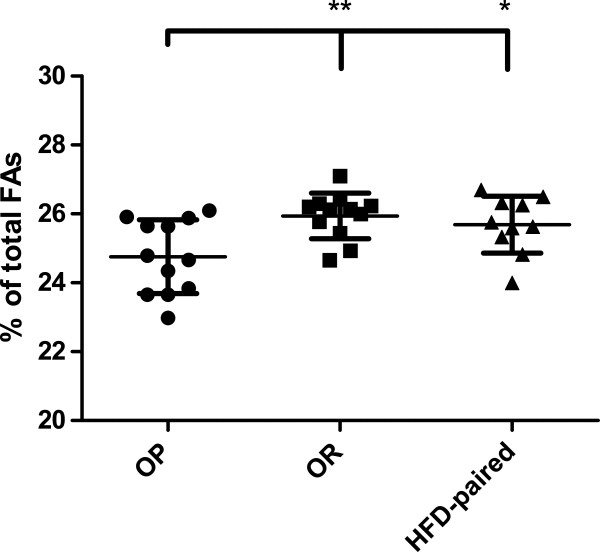
**The proportion of linoleic acid in SAT-TG decreased in OP rats fed a HFD. **HFD-induced linoleic acid (18:2n-6) proportions in subcutaneous adipose tissue triacylglycerols (SAT-TG) as relative percentages of total fatty acids (FAs). Obesity-prone (OP) rats had a significantly lower proportion than obesity-resistant (OR; P < 0.01) and HFD-paired (P < 0.05) rats. Scatter plot lines with error bars indicate means ± SD; data analyzed with One-way ANOVA and Tukey’s post-hoc test.

In SAT-TG, 18:2n-6 (linoleic acid, LA) was found to be significantly lower in OP compared with OR (P < 0.01) (Figure 3) and HFD-paired rats (P < 0.05), but there was no significant difference in LA proportions between OR and HFD-paired rats. SAT-TG 18:3n-3 (alpha-linolenic acid, ALA) was significantly higher in both OP and OR (P < 0.01) compared to HFD-paired rats. The product of SCD-1, 16:1n-7, was significantly higher in OP compared with OR (P < 0.01) and HFD-paired (P < 0.05) rats. The proportion of 22:6n-3 (docosahexaenoic acid, DHA) was significantly higher in OP compared with HFD-paired (P < 0.05), but not OR, rats. All FA comparisons can be found in Table 2.

In PL-CE, ALA was significantly lower in both OP and OR compared with HFD-paired rats (P < 0.05). While there were no differences in the 20:5n-3 (eicosapentaenoic acid, EPA) proportion between OP and HFD-paired rats, OR rats had a significantly lower proportion than HFD-paired (P < 0.05). In PL-PL, DHA was significantly higher in both OP and OR compared with HFD-paired rats (P < 0.05). In liver PL, the only significant difference was seen for DHA, of which OR had a higher proportion than HFD-paired rats (P < 0.01), which did not however differ significantly from OP rats. We were not able to statistically analyze ALA in liver PL due to a lack of data. Finally, no significant differences were found for liver TG or for any other of the studied FAs.

### Glucometabolic and adipokine levels in OP, OR and HFD-paired rats

Plasma insulin and leptin concentrations were significantly higher in OP compared with OR and HFD-paired rats, but there were no significant differences between OR and HFD-paired rats. No significant differences were observed for fasting plasma glucose values. Plasma adiponectin concentrations were significantly higher in OP compared to HFD-paired, but not OR, rats. All glucometabolic and adipokine values are shown in Table 1.

## Discussion

**Figure 4:**
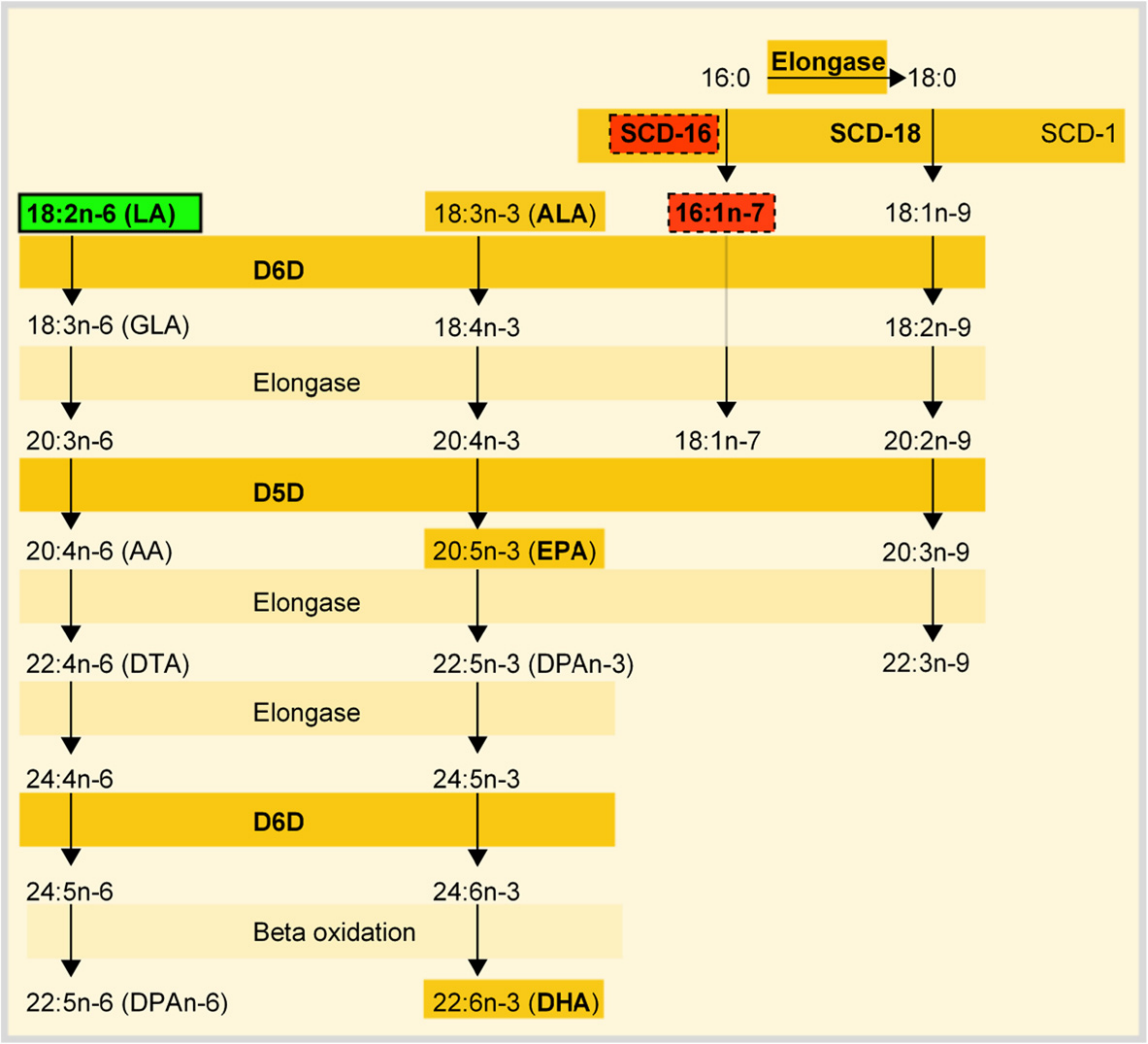
**Overview of fatty acid synthesis and HFD-induced changes between OP and OR rats. **Studied fatty acid (FA) indices and FAs have been marked bold in orange boxes. In response to a HFD, SCD-16 and 16:1n-7 in SAT-TG was higher in OP compared with OR rats (dashed bold-lined red boxes), whereas LA in SAT-TG was lower in OP compared with OR rats (solid bold-lined green box). AA, arachidonic acid; ALA, alpha-linolenic acid; D5D, delta-5 desaturase; D6D, delta-6 desaturase; DHA, docosahexaenoic acid; DPA, docosapentaenoic acid; DTA, docosatetraenoic acid; EPA, eicosapentaenoic acid, GLA, gamma-linolenic acid; HFD, high-fat diet; LA, linoleic acid; OP, obesity-prone; OR, obesity-resistant; SAT-TG, subcutaneous adipose tissue triacylglycerols; SCD-1, stearoyl-CoA desaturase 1.

In this study we examined the desaturase indices and FA composition of two different phenotypes, OP and OR, in five metabolically important lipid fractions in an outbred rat model suitable for modeling human DIO. We discovered a significant difference between OP and OR in the SCD-1 index SCD-16 and the proportion of linoleic acid in subcutaneous adipose tissue (see Figure 4 for overview), paralleled by hormonal changes indicating an insulin resistant state. Interestingly, in other lipid fractions, no significant differences between the two phenotypes were detected. This indicates that adipose tissue is affected early in the development of obesity, and alterations in the FA composition of the adipose tissue may precede or be independent of those in the liver or plasma, as supported by the observed opposing changes in the elongation index of subcutaneous adipose tissue triacylglycerols and plasma phospholipids.

Our results help to further elucidate the role that SCD-1 plays in driving susceptibility to diet-induced obesity, as it has not yet been determined whether differences in SCD-1 between OP and OR rats exist in the adipose tissue and whether these changes occur simultaneously across lipid fractions. It has previously been found that a HFD, especially a diet rich in SFA, decreases SCD expression in both rat liver and adipose tissue [33,34]. A HFD has also been shown to decrease the index SCD-16 in adipose tissue in a sex-independent manner [35]. Another study has instead shown that SFA are essential for increasing SCD-1 in mice [36], perhaps by counteracting the effect of PUFAs [37]. Human intervention studies have also reported increased SCD-16 on a SFA-rich diet [38]. It has also been found that hepatic SCD-1 levels are higher in OP versus OR rats [13] and OP versus OR mouse strains [39]. These diverging results may be due to differences in e.g. study design, diet, animal species and tissue investigated.

The higher adipose tissue SCD-16 index in OP rats might reflect a genetically inherent difference in FA metabolism pathways that, at least at an early point in DIO, is only found in this specific tissue. In a previous study we found a genetic variant of the SCD-1 gene to be associated with the degree of abdominal obesity and insulin sensitivity in elderly men [40]. In DIO, OP rats may induce SCD-1 to protect them from excess SFA storage. In this case, increased SCD-1 could perhaps be used as a marker for increased fat preference and fat intake. Interestingly, a protective role for SCD-1 could be supported by our finding that the SCD-1 product palmitoleic acid (16:1n-7) was higher in the SAT-TG in OP compared with OR rats, as this FA has been reported to serve as an insulin-sensitizing lipokine in the adipose tissue [41]. Alternatively, OR rats may downregulate SCD-1, to prevent adipose tissue storage of FAs and promote oxidation, which may be increased in OR rats [13,14]. If SCD-1 downregulation is protective against DIO, a higher activity could imply that such protective pathways are genetically dysregulated in OP animals. It has been shown that loss of SCD-1 both prevents obesity [19,42,43] and reduces inflammation in adipocytes [44]. A possible target in DIO could therefore be to selectively downregulate SCD-1 in adipose tissue, since a global knock-down of SCD-1 has been shown to lead to deleterious effects [4].

The non-uniform changes in SCD-16 activity across lipid fractions in this study, could be due to differences in species, strain and experimental duration: Li *et al.* used Wistar rats exposed to a 16-week HFD – notably longer than our 5-week treatment, which may therefore reflect earlier changes – while Hu *et al.* fed obesity-prone (C57BL/6) and obesity-resistant (FVB) mouse strains a HFD for 8 weeks [13,39]. Both of the aforementioned studies examined SCD-1 mRNA expression, whereas we studied the indices SCD-16 and SCD-18. In our study, the lack of difference in SCD-18, an index also employed to reflect SCD-1 enzyme activity, could be due to the high dietary content of the SCD-18 product oleic acid (OA; 18:1n-9), which could influence the SCD-18 index [31]. At least in our DIO model, the adipose tissue may be one of the first tissues affected by DIO. This was evident as an altered FA metabolism with up-regulated SCD-1 activity index and decreased linoleic acid proportions in OP rats. Changes in the liver, such as those observed in other studies, might then follow as insulin-resistance worsens. This further highlights the importance of the adipose tissue as a key metabolic tissue involved in the development of obesity.

We also found that OP rats had a significantly lower proportion of linoleic acid in SAT-TG, both compared to OR and the calorically restricted rats. Low proportions of LA in humans have previously been found in plasma of obese subjects [27]. In adipose tissue of older men, LA has recently been weakly but positively correlated with insulin sensitivity [45]. Intervention studies suggest that LA-rich diets may decrease SAT and improve insulin sensitivity [46], in line with a possible anti-diabetic effect of LA [47] that should be further investigated.

The higher SCD-1 in OP compared with OR rats could be due to the higher insulin levels seen in the OP rats, as insulin is known to regulate SCD-1 transcription [42,48]. Such interpretation is on the other hand complicated by the elevated leptin concentrations observed in OP rats, as leptin has been shown to decrease both hepatic and adipose tissue SCD-1 expression [49,50]. No difference in adiponectin levels were however found between OP and OR rats. We however did find that plasma adiponectin levels were higher in OP compared with HFD-paired rats, which may be due to an increased adiponectin resistance in OP compared with HFD-paired rats, with an at least initial compensatory higher adiponectin production [51-54].

OP but not OR rats showed a correlation between food intake and dBw. This strengthens the notion that OR rats have biochemical differences rendering them less efficient at converting food into fat tissue. This difference could perhaps simply be caused by a greater genetic heterogeneity within the OR group. It has however been discovered that on a HFD, OR rats seem to have a proportionately higher fat oxidation [14], and that OR in male but not female rats is associated with better food intake control [9]. Furthermore, a transcriptomic analysis previously found that OP rats have differences in metabolic pathways involving the Krebs cycle, increased ketone body production and a cholesterol transfer promoting fat storage [13]. Our finding that OR rats had a lower food efficiency than OP rats is supported by the findings reported by Chang *et al.*[14]. The two phenotypes seen in this study therefore seem to present two other major differences: a reduced food intake in OR rats, possibly due to a more optimal reward system [55], and a reduced food efficiency, perhaps linked to lower SCD-1 activity or other biochemical differences in the handling of FAs.

Since we had a control group that was calorically restricted but fed an identical HFD as the one fed ad libitum to the OP and OR groups, we could evaluate the effects of both ad libitum HFD intake and obesity phenotype on FA metabolism. The SCD-16 index of the SAT-TG was significantly higher in OP rats compared with the energy-restricted HFD-paired rats, whereas there was no difference between the OR and HFD-paired rats. Thus, even after moderate weight gain and ad libitum access to the HFD, the OR rats seem to be metabolically similar to the HFD-paired rats. However, for most of the desaturase indices and FAs no differences were observed when ad-libitum-fed rats were compared with the calorically restricted rats, suggesting that on a HFD, many of these parameters are weight-independent.

It should be noted that the diets used to induce DIO more than often differ between different studies [21], thus complicating comparisons between studies. The FA composition of the diet could mask potential differences in FA composition between OP and OR rats that would have been seen if the animals had been given a different diet. However, on the other hand, both the calorie-restricted and the ad libitum groups received the same diet, making the results more attributable to metabolic differences. To elucidate what drives the OP and OR phenotypes, initial measurements of the FA composition would have been valuable. Such data could have provided additional predictive markers for susceptibility to DIO; some of which, e.g. plasma-derived FA, could be easily measurable in human subjects. E.g. SCD-1 is known to be correlated with markers of obesity [27], but it is still unknown whether these pathological changes are caused by SCD-1 per se, or if it is a marker of or perhaps even a compensatory mechanism for these ongoing processes [31]. Furthermore, the adipose tissue analyzed herein was derived from the inguinal fat depot, and does not necessarily reflect other adipose tissue depots, as reported by other researchers [35,56].

## Conclusion

In this experiment we demonstrate that obesity-prone rats have a significantly higher SCD-16 index, significantly higher proportions of the SCD-1 product 16:1n-7, but a lower proportion of LA in adipose tissue. These changes are largely independent of weight gain and seem to appear before any changes in the plasma or liver tissue, and are paralleled by hormonal changes indicating insulin resistance with possible compensatory changes. Whether a lower SCD-16 index protects against DIO is an interesting possibility that warrants further investigation.

## Methods

Thirty-four male outbred Sprague–Dawley rats (Scanbur B&K, Sollentuna, Sweden) were used in this study. At the start of the experiments the rats were 8 weeks old and weighed 352 ± 12 g (mean ± SD). The rats were housed one per cage, in standard macrolon cages (type IV), which had a wood chip bedding and a wooden house as enrichment. All rats had free access to water*.* Ambient temperature (21-22°C) and humidity (40–50%) were kept constant and a 12-h-light cycle with lights on at 07:00 was used. The rats were allowed one week of adaptation to the animal facility conditions before onset of the experiments. The rats were then randomly divided into two dietary groups. One group of rats were fed a HFD ad libitum (AL-HFD, n = 24); the control group (HFD-paired, n = 10) were fed the HFD but were calorically restricted to follow the weight trajectory of male Sprague–Dawley rats with ad libitum access to regular chow. The HFD (D12451, ResearchDiets) contained 19.79 kJ (4.73 kcal)/g (20% protein, 35% carbohydrates and 45% fat by energy; see Table 3 for composition). During the five weeks of access to the diets, food intake and body weight were measured daily during the first week and thereafter three times a week. After the five-week dietary intervention, the rats were fasted for three hours and were then killed by decapitation, at which point trunk blood was collected. Plasma was isolated by centrifugation and stored at −80°C until analyzed. Liver tissue and subcutaneous adipose tissue (SAT) from the inguinal region were isolated and stored at −80°C. Note that data from a subset of these rats have previously been analyzed and published [56].

**Table 3:** Dietary composition of the experimental diet

**Composition by weight (g/kg)**	
Casein, 80 Mesh	233
L-Cystine	3.5
Corn Starch	84.8
Maltodextrin	116.5
Sucrose	201.4
Cellulose, BW200	58.3
Soybean Oil	29.1
Lard	206.8
Mineral Mix S10026	11.7
DiCalcium Phosphate	15.1
Calcium Carbonate	6.4
Potassium Citrate, 1 H2O	19.2
Vitamin Mix V10001	11.7
Choline Bitartrate	2.3
FD&C Red Dye #40	0.06
**FA profile (% of total FAs)**	
Saturated	36.3
Monounsaturated	45.3
Polyunsaturated	18.5

### Analysis of fatty acid composition

Gas chromatography was used as previously described by Boberg *et al.*[57] to analyze the FA composition of five different lipid fractions: subcutaneous adipose tissue triacylglycerols (SAT-TG), plasma cholesterol esters (PL-CE), plasma phospholipids (PL-PL), liver phospholipids (liver PL) and liver triacylglycerols (liver TG).

For the SAT and liver tissue, each tissue (5-25 mg of SAT; 40–50 mg of liver tissue) was extracted in 2.5 ml methanol and 5.0 ml chloroform (containing 0.005% butylated hydroxytoluene as an antioxidant). 7.5 ml 0.2 mol/l sodium dihydrogen phosphate (Na_2_H_2_PO_4_) was added and the extract was left at +4°C over night. Both the liver and SAT chloroform phases were evaporated to dryness under nitrogen. The SAT lipid esters were transmethylated at 60°C overnight after addition of 2 ml of 5% H_2_SO_4_ in methanol.

For lipid extraction from plasma, 2.5 ml of methanol was added to 0.5 ml of plasma. After thorough mixing of the extract, 5.0 ml Chloroform (containing 0.005% butylated hydroxytoluene as an antioxidant) and 7.5 ml of 0.2 mM sodium dihydrogen phosphate (Na_2_H_2_PO_4_) were added and were left at + 4°C over night. The chloroform phase was evaporated to dryness under nitrogen and the lipid residue was dissolved in 200–300 μl chloroform.

For the plasma and liver lipid fractions, the lipid esters (triacylglycerols and phospholipids from liver; cholesterol esters and phospholipids from plasma) were separated by thin-layer chromatography (TLC). The chloroform solution was applied to the adsorbent (Silica gel 60G, Merck) containing POPOP as a fluorescent agent. The TLC plates were eluted at room temperature with the solvent system petroleum benzine/diethyl ether/acetic acid (81:18:1, by vol). The lipid fractions were visualized in UV light and the spots were scraped off into vials and the lipid esters were transmethylated at 60°C overnight after addition of 2 ml 5% H_2_SO_4_ in methanol.

For all five lipid fractions, the methyl esters were extracted into 3 ml of petroleum ether containing 0.005% butylated hydroxytolvene after addition of 1.5 ml distilled water. The phases were separated after thorough mixing and centrifugation at 1500 × g for 10 min. The petroleum ether phase was pipetted off and the solvent was evaporated under nitrogen. The methyl esters were then redissolved in Uvasol, grade hexane.

The FA methyl esters from the five different fractions were separated by gas chromatography (GC) on a 30-m glass capillary column coated with Thermo TR-FAME (Thermo Electron Corporation, USA), with helium gas as a carrier gas. An Agilent Technologies system consisting of model GC 6890N, autosampler 7683 and Agilent ChemStation was used. The temperature was programmed to 150–260°C. The FAs were identified by comparing each peak’s retention time with FA methyl ester standard Nu Check Prep (Elysian, MN, USA) and are expressed as relative percentages of total FAs. Due to low proportions, not all FAs were quantifiable in all samples.

### Glucometabolic and adipokine analyses

Plasma insulin and glucose were analyzed with validated in-house methods, whereas plasma adiponectin and leptin were measured with RIA kits according to the manufacturer's instructions (Millipore AB, Solna, Sweden).

### Statistical analyses

To minimize the number of statistical tests, we chose to limit our statistical tests of FAs to the indices for desaturases (SCD-16 (16:1n-7/16:0), SCD-18 (18:1n-9/18:0), D6D (18:3n-6/18:2n-6), D5D (20:4n-6/20:3n-6), de novo lipogenesis (DNL; 16:0/18:2n-6) and elongation (EI, 18:0/16:0), and important polyunsaturated FAs with bioactive properties, i.e. essential n-6 (18:2n-6, LA), and n-3 (18:3n-3, ALA) FAs as well as very long chain n-3 FAs (20:5n-3, EPA; 22:6n-3, DHA). We also investigated the proportions of the SCD-16 product, 16:1n-7 (palmitoleate), since 16:1n-7 has been proposed as an adipokine [41].

GraphPad Prism v 5.02 (GraphPad Software Inc., San Diego, CA) was used for all statistical calculations. Kolmogorov-Smirnov’s test of normality was first used to determine whether data was normally distributed. The following parameters were found to be non-normally distributed in OP rats: SAT-TG D6D; PL-CE DNL index and EI; liver PL SCD-18, D6D and D5D; and liver TG SCD-16, DNL index, EI, 16:1n-7, and LA. In OR rats the non-normally distributed parameters were: dBw; SAT-TG D6D; PL-CE LA; and PL-PL EI. In HFD-paired rats the non-normally distributed parameters were: initial body weight; plasma insulin concentrations; SAT-TG D6D and ALA; PL-PL ALA and EPA; and liver TG DHA. To detect differences between the three different groups, the investigated normally and non-normally distributed parameters were analyzed using, respectively, One-way ANOVA or Kruskal-Wallis ANOVA. For significant ANOVAs, we proceeded with post-hoc tests to find significant group differences: for One-way ANOVA, Tukey’s test was used; for Kruskal-Wallis, Dunn’s test was used. When only two groups were compared, the Student’s *t*-test was used for normally distributed data. For correlations, non-normally distributed data was analyzed using Spearman’s rank correlation. Values were considered significant at the P < 0.05 level and are presented as means ± SD.

### Ethical statement

The Ethical Committee for Animal Experiments in Uppsala approved the experiments and procedures. Animal care procedures followed guidelines of EU (Convention ETS123 and Directive86/609/EEC) and Swedish (Animal Welfare Act SFS1998:56) legislation on animal experiments.

## Abbreviations

AL-HFD: Ad libitum high-fat diet; ALA: Alpha-linolenic acid; LA: Linoleic acid; D5D: Delta-5 desaturase; D6D: Delta-6 desaturase; DHA: Docosahexaenoic acid; dBw: Difference (gain) in body weight; DNL: De novo lipogenesis (index); EI: Elongation index; EPA: Eicosapentaenoic acid; FA: Fatty acid; HFD: High-fat diet; LA: Linoleic acid; OP: Obesity prone; OR: Obesity resistant; PL-CE: Plasma cholesterol esters; PL: Phospholipids; PL-PL: Plasma phospholipids; SAT-TG: Subcutaneous adipose tissue triacylglycerols; SCD: Stearoyl-CoA desaturase; SCD-1: Stearoyl-CoA desaturase 1; TG: Triacylglycerols.

## Competing interests

The authors declare that they have no competing interests.

## Authors’ contributions

J. C. was responsible for analyzing and interpreting the data and writing the manuscript; J. A. was responsible for planning and conducting the experiment, analyzing the data, and writing the manuscript; Å. V. was responsible for analyzing the data, and writing the manuscript; U. R. was responsible for interpreting the data and writing the manuscript; H. B. S. was responsible for planning the study, interpreting the data and writing the manuscript. All authors read and approved the final manuscript.

## References

[B1] Bessesen DH Bull S Cornier MA Trafficking of dietary fat and resistance to obesity Physiol Behav 2008 94 681 688 10.1016/j.physbeh.2008.04.019 18514237 PMC2494849

[B2] Blundell JE Stubbs RJ Golding C Croden F Alam R Whybrow S Le Noury J Lawton CL Resistance and susceptibility to weight gain: individual variability in response to a high-fat diet Physiol Behav 2005 86 614 622 10.1016/j.physbeh.2005.08.052 16225895

[B3] Flatt JP What do we most need to learn about food intake regulation? Obes Res 1998 6 307 310 10.1002/j.1550-8528.1998.tb00354.x 9688108

[B4] Pickering C Alsio J Hulting AL Schioth HB Withdrawal from free-choice high-fat high-sugar diet induces craving only in obesity-prone animals Psychopharmacology (Berl) 2009 204 431 443 10.1007/s00213-009-1474-y 19205668

[B5] Schemmel R Mickelsen O Gill JL Dietary obesity in rats: Body weight and body fat accretion in seven strains of rats J Nutr 1970 100 1041 1048 5456549 10.1093/jn/100.9.1041

[B6] West DB Boozer CN Moody DL Atkinson RL Dietary obesity in nine inbred mouse strains Am J Physiol 1992 262 R1025 R1032 1621856 10.1152/ajpregu.1992.262.6.R1025

[B7] Levin BE Triscari J Hogan S Sullivan AC Resistance to diet-induced obesity: food intake, pancreatic sympathetic tone, and insulin Am J Physiol 1987 252 R471 R478 3548441 10.1152/ajpregu.1987.252.3.R471

[B8] Levin BE Triscari J Sullivan AC Relationship between sympathetic activity and diet-induced obesity in two rat strains Am J Physiol 1983 245 R364 R371 6614206 10.1152/ajpregu.1983.245.3.R364

[B9] Jackman MR MacLean PS Bessesen DH Energy expenditure in obesity-prone and obesity-resistant rats before and after the introduction of a high-fat diet Am J Physiol Regul Integr Comp Physiol 2010 299 R1097 R1105 10.1152/ajpregu.00549.2009 20686168 PMC2957378

[B10] Dobrian AD Davies MJ Prewitt RL Lauterio TJ Development of hypertension in a rat model of diet-induced obesity Hypertension 2000 35 1009 1015 10.1161/01.HYP.35.4.1009 10775577

[B11] Perez-Echarri N Noel-Suberville C Redonnet A Higueret P Martinez JA Moreno-Aliaga MJ Role of adipogenic and thermogenic genes in susceptibility or resistance to develop diet-induced obesity in rats J Physiol Biochem 2007 63 317 327 10.1007/BF03165763 18457007

[B12] Wang X Choi JW Joo JI Kim DH Oh TS Choi DK Yun JW Differential expression of liver proteins between obesity-prone and obesity-resistant rats in response to a high-fat diet Br J Nutr 2011 106 612 626 10.1017/S0007114511000651 21535901

[B13] Li H Xie Z Lin J Song H Wang Q Wang K Su M Qiu Y Zhao T Song K Transcriptomic and metabonomic profiling of obesity-prone and obesity-resistant rats under high fat diet J Proteome Res 2008 7 4775 4783 10.1021/pr800352k 18828625

[B14] Chang S Graham B Yakubu F Lin D Peters JC Hill JO Metabolic differences between obesity-prone and obesity-resistant rats Am J Physiol 1990 259 R1103 R1110 2260721 10.1152/ajpregu.1990.259.6.R1103

[B15] Ji H Friedman MI Reduced capacity for fatty acid oxidation in rats with inherited susceptibility to diet-induced obesity Metabolism 2007 56 1124 1130 10.1016/j.metabol.2007.04.006 17618960 PMC1995409

[B16] Ji H Outterbridge LV Friedman MI Phenotype-based treatment of dietary obesity: differential effects of fenofibrate in obesity-prone and obesity-resistant rats Metabolism 2005 54 421 429 10.1016/j.metabol.2004.10.007 15798946

[B17] Jackman MR Kramer RE MacLean PS Bessesen DH Trafficking of dietary fat in obesity-prone and obesity-resistant rats Am J Physiol Endocrinol Metab 2006 291 E1083 E1091 10.1152/ajpendo.00159.2006 16803858

[B18] Abu-Elheiga L Matzuk MM Abo-Hashema KA Wakil SJ Continuous fatty acid oxidation and reduced fat storage in mice lacking acetyl-CoA carboxylase 2 Science 2001 291 2613 2616 10.1126/science.1056843 11283375

[B19] Ntambi JM Miyazaki M Stoehr JP Lan H Kendziorski CM Yandell BS Song Y Cohen P Friedman JM Attie AD Loss of stearoyl-CoA desaturase-1 function protects mice against adiposity Proc Natl Acad Sci U S A 2002 99 11482 11486 10.1073/pnas.132384699 12177411 PMC123282

[B20] Moussavi N Gavino V Receveur O Could the quality of dietary fat, and not just its quantity, be related to risk of obesity? Obesity (Silver Spring) 2008 16 7 15 10.1038/oby.2007.14 18223605

[B21] Hariri N Thibault L High-fat diet-induced obesity in animal models Nutr Res Rev 2010 23 270 299 10.1017/S0954422410000168 20977819

[B22] Kawashima A Sugawara S Okita M Akahane T Fukui K Hashiuchi M Kataoka C Tsukamoto I Plasma fatty acid composition, estimated desaturase activities, and intakes of energy and nutrient in Japanese men with abdominal obesity or metabolic syndrome J Nutr Sci Vitaminol (Tokyo) 2009 55 400 406 10.3177/jnsv.55.400 19926925

[B23] Vessby B Gustafsson IB Tengblad S Boberg M Andersson A Desaturation and elongation of Fatty acids and insulin action Ann N Y Acad Sci 2002 967 183 195 12079847 10.1111/j.1749-6632.2002.tb04275.x

[B24] Warensjo E Riserus U Vessby B Fatty acid composition of serum lipids predicts the development of the metabolic syndrome in men Diabetologia 2005 48 1999 2005 10.1007/s00125-005-1897-x 16132958

[B25] Warensjo E Sundstrom J Vessby B Cederholm T Riserus U Markers of dietary fat quality and fatty acid desaturation as predictors of total and cardiovascular mortality: a population-based prospective study Am J Clin Nutr 2008 88 203 209 18614742 10.1093/ajcn/88.1.203

[B26] Kotronen A Seppanen-Laakso T Westerbacka J Kiviluoto T Arola J Ruskeepaa AL Oresic M Yki-Jarvinen H Hepatic stearoyl-CoA desaturase (SCD)-1 activity and diacylglycerol but not ceramide concentrations are increased in the nonalcoholic human fatty liver Diabetes 2009 58 203 208 10.2337/db08-1074 18952834 PMC2606873

[B27] Warensjo E Ohrvall M Vessby B Fatty acid composition and estimated desaturase activities are associated with obesity and lifestyle variables in men and women Nutr Metab Cardiovasc Dis 2006 16 128 136 10.1016/j.numecd.2005.06.001 16487913

[B28] Peter A Cegan A Wagner S Lehmann R Stefan N Konigsrainer A Konigsrainer I Haring HU Schleicher E Hepatic lipid composition and stearoyl-coenzyme A desaturase 1 mRNA expression can be estimated from plasma VLDL fatty acid ratios Clin Chem 2009 55 2113 2120 10.1373/clinchem.2009.127274 19850634

[B29] Sampath H Ntambi JM Polyunsaturated fatty acid regulation of gene expression Nutr Rev 2004 62 333 339 10.1111/j.1753-4887.2004.tb00058.x 15497766

[B30] Jump DB Botolin D Wang Y Xu J Christian B Demeure O Fatty acid regulation of hepatic gene transcription J Nutr 2005 135 2503 2506 16251601 10.1093/jn/135.11.2503

[B31] Bjermo H Riserus U Role of hepatic desaturases in obesity-related metabolic disorders Curr Opin Clin Nutr Metab Care 2010 13 703 708 10.1097/MCO.0b013e32833ec41b 20823776

[B32] Flowers MT Ntambi JM Stearoyl-CoA desaturase and its relation to high-carbohydrate diets and obesity Biochim Biophys Acta 2009 1791 85 91 10.1016/j.bbalip.2008.12.011 19166967 PMC2649790

[B33] Kakuma T Lee Y Unger RH Effects of leptin, troglitazone, and dietary fat on stearoyl CoA desaturase Biochem Biophys Res Commun 2002 297 1259 1263 10.1016/S0006-291X(02)02375-6 12372423

[B34] Qiu J Cheng R Zhou XY Zhu JG Zhu C Qin DN Kou CZ Guo XR Gene expression profiles of adipose tissue of high-fat diet-induced obese rats by cDNA microarrays Mol Biol Rep 2010 37 3691 3695 10.1007/s11033-010-0021-6 20191385

[B35] Estrany ME Proenza AM Llado I Gianotti M Isocaloric intake of a high-fat diet modifies adiposity and lipid handling in a sex dependent manner in rats Lipids Health Dis 2011 10 52 10.1186/1476-511X-10-52 21486445 PMC3095551

[B36] Sampath H Miyazaki M Dobrzyn A Ntambi JM Stearoyl-CoA desaturase-1 mediates the pro-lipogenic effects of dietary saturated fat J Biol Chem 2007 282 2483 2493 17127673 10.1074/jbc.M610158200

[B37] Ntambi JM Miyazaki M Recent insights into stearoyl-CoA desaturase-1 Curr Opin Lipidol 2003 14 255 261 10.1097/00041433-200306000-00005 12840656

[B38] Warensjo E Riserus U Gustafsson IB Mohsen R Cederholm T Vessby B Effects of saturated and unsaturated fatty acids on estimated desaturase activities during a controlled dietary intervention Nutr Metab Cardiovasc Dis 2008 18 683 690 10.1016/j.numecd.2007.11.002 18367385

[B39] Hu CC Qing K Chen Y Diet-induced changes in stearoyl-CoA desaturase 1 expression in obesity-prone and -resistant mice Obes Res 2004 12 1264 1270 10.1038/oby.2004.160 15340109

[B40] Warensjo E Ingelsson E Lundmark P Lannfelt L Syvanen AC Vessby B Riserus U Polymorphisms in the SCD1 gene: associations with body fat distribution and insulin sensitivity Obesity (Silver Spring) 2007 15 1732 1740 10.1038/oby.2007.206 17636091

[B41] Cao H Gerhold K Mayers JR Wiest MM Watkins SM Hotamisligil GS Identification of a lipokine, a lipid hormone linking adipose tissue to systemic metabolism Cell 2008 134 933 944 10.1016/j.cell.2008.07.048 18805087 PMC2728618

[B42] Cohen P Miyazaki M Socci ND Hagge-Greenberg A Liedtke W Soukas AA Sharma R Hudgins LC Ntambi JM Friedman JM Role for stearoyl-CoA desaturase-1 in leptin-mediated weight loss Science 2002 297 240 243 10.1126/science.1071527 12114623

[B43] Flowers JB Rabaglia ME Schueler KL Flowers MT Lan H Keller MP Ntambi JM Attie AD Loss of stearoyl-CoA desaturase-1 improves insulin sensitivity in lean mice but worsens diabetes in leptin-deficient obese mice Diabetes 2007 56 1228 1239 10.2337/db06-1142 17369521

[B44] Liu X Miyazaki M Flowers MT Sampath H Zhao M Chu K Paton CM Joo DS Ntambi JM Loss of Stearoyl-CoA desaturase-1 attenuates adipocyte inflammation: effects of adipocyte-derived oleate Arterioscler Thromb Vasc Biol 2010 30 31 38 10.1161/ATVBAHA.109.195636 19910642 PMC2837593

[B45] Iggman D Arnlov J Vessby B Cederholm T Sjogren P Riserus U Adipose tissue fatty acids and insulin sensitivity in elderly men Diabetologia 2010 53 850 857 10.1007/s00125-010-1669-0 20127308

[B46] Summers LK Fielding BA Bradshaw HA Ilic V Beysen C Clark ML Moore NR Frayn KN Substituting dietary saturated fat with polyunsaturated fat changes abdominal fat distribution and improves insulin sensitivity Diabetologia 2002 45 369 377 10.1007/s00125-001-0768-3 11914742

[B47] Riserus U Willett WC Hu FB Dietary fats and prevention of type 2 diabetes Prog Lipid Res 2009 48 44 51 10.1016/j.plipres.2008.10.002 19032965 PMC2654180

[B48] Hodson L Fielding BA Stearoyl-CoA desaturase: rogue or innocent bystander? Prog Lipid Res 2012 52 15 42 23000367 10.1016/j.plipres.2012.08.002

[B49] Lin J Choi YH Hartzell DL Li C Della-Fera MA Baile CA CNS melanocortin and leptin effects on stearoyl-CoA desaturase-1 and resistin expression Biochem Biophys Res Commun 2003 311 324 328 10.1016/j.bbrc.2003.10.004 14592417

[B50] Zhang W Della-Fera MA Hartzell DL Hausman D Baile CA Adipose tissue gene expression profiles in ob/ob mice treated with leptin Life Sci 2008 83 35 42 10.1016/j.lfs.2008.04.021 18547592

[B51] Mullen KL Pritchard J Ritchie I Snook LA Chabowski A Bonen A Wright D Dyck DJ Adiponectin resistance precedes the accumulation of skeletal muscle lipids and insulin resistance in high-fat-fed rats Am J Physiol Regul Integr Comp Physiol 2009 296 R243 R251 19073900 10.1152/ajpregu.90774.2008

[B52] Oana F Takeda H Hayakawa K Matsuzawa A Akahane S Isaji M Akahane M Physiological difference between obese (fa/fa) Zucker rats and lean Zucker rats concerning adiponectin Metabolism 2005 54 995 1001 10.1016/j.metabol.2005.02.016 16092047

[B53] Perez-Echarri N Perez-Matute P Martinez JA Marti A Moreno-Aliaga MJ Serum and gene expression levels of leptin and adiponectin in rats susceptible or resistant to diet-induced obesity J Physiol Biochem 2005 61 333 342 10.1007/BF03167050 16180331

[B54] Bullen JW Jr Bluher S Kelesidis T Mantzoros CS Regulation of adiponectin and its receptors in response to development of diet-induced obesity in mice Am J Physiol Endocrinol Metab 2007 292 E1079 E1086 17164441 10.1152/ajpendo.00245.2006

[B55] Alsio J Olszewski PK Norback AH Gunnarsson ZE Levine AS Pickering C Schioth HB Dopamine D1 receptor gene expression decreases in the nucleus accumbens upon long-term exposure to palatable food and differs depending on diet-induced obesity phenotype in rats Neuroscience 2010 171 779 787 10.1016/j.neuroscience.2010.09.046 20875839

[B56] Caesar R Manieri M Kelder T Boekschoten M Evelo C Muller M Kooistra T Cinti S Kleemann R Drevon CA A combined transcriptomics and lipidomics analysis of subcutaneous, epididymal and mesenteric adipose tissue reveals marked functional differences PLoS One 2010 5 e11525 10.1371/journal.pone.0011525 20634946 PMC2902507

[B57] Boberg M Croon LB Gustafsson IB Vessby B Platelet fatty acid composition in relation to fatty acid composition in plasma and to serum lipoprotein lipids in healthy subjects with special reference to the linoleic acid pathway Clin Sci (Lond) 1985 68 581 587 3919990 10.1042/cs0680581

